# ICOS is upregulated on T cells following radiation and agonism combined with radiation results in enhanced tumor control

**DOI:** 10.1038/s41598-022-19256-8

**Published:** 2022-09-02

**Authors:** Tiffany Blair, Jason Baird, Shelly Bambina, Gwen Kramer, Monica Gostissa, Christopher J. Harvey, Michael J. Gough, Marka R. Crittenden

**Affiliations:** 1 Earle A. Chiles Research Institute, Robert W. Franz Cancer Center, Providence Portland Medical Center, 4805 NE Glisan St, North Pavilion, Suite 2N108, Portland, OR 97213 USA; 2 Jounce Therapeutics, Inc., 780 Memorial Drive, Cambridge, MA 02139 USA; 3 The Oregon Clinic, Portland, OR 97213 USA; 4 Present Address: Phenomic AI, 661 University Ave Suite 1300, Toronto, ON M5G 0B7 Canada

**Keywords:** Cancer, Immunology, Oncology

## Abstract

Multiple preclinical studies have shown improved outcomes when radiation therapy is combined with immune modulating antibodies. However, to date, many of these promising results have failed to translate to successful clinical studies. This led us to explore additional checkpoint and co-stimulatory pathways that may be regulated by radiation therapy. Here, we demonstrate that radiation increases the expression of inducible T cell co-stimulator (ICOS) on both CD4 and CD8 T cells in the blood following treatment. Moreover, when we combined a novel ICOS agonist antibody with radiation we observed durable cures across multiple tumor models and mouse strains. Depletion studies revealed that CD8 T cells were ultimately required for treatment efficacy, but CD4 T cells and NK cells also partially contributed to tumor control. Phenotypic analysis showed that the combination therapy diminished the increased infiltration of regulatory T cells into the tumor that typically occurs following radiation alone. Finally, we demonstrate in a poorly immunogenic pancreatic tumor model which is resistant to combined radiation and anti-PD1 checkpoint blockade that the addition of this novel ICOS agonist antibody to the treatment regimen results in tumor control. These findings identify ICOS as part of a T cell pathway that is modulated by radiation and targeting this pathway with a novel ICOS antibody results in durable tumor control in preclinical models.

## Introduction

Extensive studies have demonstrated that radiation therapy results in tumor control that is dependent on T cells in preclinical tumor models. In preclinical models, some portion of the immune response against tumors is generated following the implantation of the cancer cells into immune competent animals^1–3^, which relates to the intrinsic immunogenicity of these tumor models^4^. The pre-existing tumor-specific T cells in the tumor can synergize with radiation therapy to kill residual cancer cells by radiation-mediated upregulation of antigen processing and presentation^5,6^. In addition, radioimmunogenic tumors can also generate new immune responses following radiation therapy via antigen loading and maturation of cross-presenting dendritic cells in the tumor^7^. By contrast, in poorly radioimmunogenic tumors, DC maturation fails to occur following radiation, limiting the efficacy of radiation as an endogenous vaccine^4,7,8^. While T cells have therefore been shown to play an important role in control of residual disease following radiation, in most preclinical models radiation therapy is not sufficient to eradicate tumors without the addition of immunotherapies to enhance the radiation-induced immune response.

T cells express a range of receptors following activation that can serve to stimulate or suppress their response to cognate antigens. Pharmacological agents that target these receptors have shown promise for clinical treatment of multiple tumor types^9,10^. Checkpoint inhibitors such as CTLA4 and PD1/PDL1 serve to relieve suppression of existing tumor-specific T cells, and these antibodies and other costimulatory antibodies also have the potential to generate new anti-tumor immune responses by supporting expansion of new tumor-specific T cells that were previously not involved in tumor control^9–12^. In preclinical models, radiation therapy has shown synergy with both agonists of costimulatory targets such as OX40 and 41BB^13–15^ as well as antagonists of co-inhibitory targets such as CTLA4 and PD1^16,17^. Unfortunately, in clinical studies, blockade of CTLA4 or PD1 in combination with radiation have yet to show significant improvement in systemic control of disease when compared to single agent immunotherapy^18–20^. At present, antibodies targeting OX40 and 41BB have not yet been combined with radiation therapy in a randomized study and the clinical efficacy of these agents remains to be determined. Given the limited clinical impact of combined radiation therapy and immunotherapy studies to date, identifying novel targets that are regulated by radiation may provide insight into alternative therapies or combination therapies that show greater promise for future clinical development.

Inducible T-cell costimulator (ICOS; CD278) is one such potential target. ICOS has been shown to be upregulated on both CD4 and CD8 T cells following activation^21^, and ligation of ICOS improves T cell proliferation and differentiation into effectors (reviewed in^22,23^). Moreover, ICOS is also highly expressed on CD4 T regulatory cells^24^ and represents a potential means to alter the suppression versus activation balance in tumors^25^. We therefore sought to investigate the regulation of ICOS expression following tumor radiation and evaluate the potential effects of combining radiation therapy with an ICOS agonist antibody in preclinical mouse tumor models.

Our data demonstrate that ICOS is upregulated on CD4 and CD8 T cells in the peripheral blood and tumor following radiation therapy, and that the combination of a novel ICOS agonist antibody with radiation therapy results in tumor control. This efficacy was observed in different tumor types and different radiation regimens. In addition, ICOS agonist antibody and radiation therapy combined with PD1/PDL1 axis blockade resulted in cures even in recalcitrant pancreatic tumor models.

## Materials and methods

### Cell lines and mice

Animal protocols were approved by the Earle A. Chiles Research Institute (EACRI) Institutional Animal Care and Use Committee (Animal Welfare Assurance No. A3913-01). All experiments were performed in accordance with relevant guidelines and regulations and is reported in accordance with ARRIVE guidelines. Experiments utilized 6–8 week old BALB/c and C57BL/6 mice that were obtained from The Jackson Laboratories. Survival experiments were performed with 6–10 mice per experimental group, and mechanistic experiments with 4–6 mice per group. The CT26 murine colorectal carcinoma cell line^26^ was obtained from ATCC (Manassas, VA). The Panc02 murine pancreatic adenocarcinoma^27^ cell line was kindly provided by Dr. Savio Woo (Mount Sinai School of Medicine, New York, NY). The Moc1 murine oral squamous cell carcinoma line^28^ was kindly provided by Dr. Ravindra Uppaluri at the Dana Faber Cancer Institute. Cell lines were grown in complete RPMI containing 10% heat inactivated fetal bovine serum (FBS), 100U/mL penicillin, 100 µg/mL streptomycin.

### Tumor implantation and treatments

Tumors were implanted subcutaneously into the right flank as follows; 2 × 10^5^ CT26, 2 × 10^5^ Panc02, and 1 × 10^6^ Moc1. At day 14, mice were randomized to receive treatment with CT-guided radiation using the Small Animal Radiation Research Platform (Xstrahl, Suwanee, GA) and Murislice software (Xstrahl) as previously described^29^. The SARRP delivered a single dose of 12 Gy to an isocenter within the tumor using a 10 mm × 10 mm collimator and a 45 degree beam angle to minimize dose delivery to normal tissues. For ICOS Ab treatment, two doses of 0.25 mg/kg (Jounce Therapeutics, clone 36E10 mouse IgG2a) were administered intraperitoneally 7 days apart, with the timing of the first dose varying depending on the experiment. Control mice received equal dosing of an isotype control antibody where stated. For PD1 antibody treatment, three doses of 250 µg of anti-PD1 antibodies from BioXCell (clone RMP1-14) were administered intraperitoneally 7 days apart.

For CD8 depletion, 250 µg of anti-CD8beta antibodies from BioXCell (clone Lyt 3.2) were given intraperitoneally one day prior to radiation and again 7 days later. For CD4 depletion, 100 µg of anti-CD4 antibodies from BioXCell (clone GK1.5) were given intraperitoneally one day prior to radiation and again 7 days later. For NK cell deption, 100 µl of anti-Asialo GM1 from Wako Chemicals were given intraperitoneally one day prior to radiation and again 7 days later. In all survival experiments, tumor length and width were measured 2–3 times per week using calipers. Mice were euthanized when tumor size exceeded 12 mm in any dimension, or when body condition score declined below 1 level.

### Blood analysis

Whole blood from euthanized mice was collected by venipuncture directly into sodium citrate (3.2% w/v) at a ratio of 9:1 v/v. Quantification of antigen-specific cell numbers in peripheral blood was measured using a whole blood assay. Blood was stained directly with fluorescent antibody cocktails as previously described^1^. AccuCheck fluorescent beads (Invitrogen) were added to each sample before lysing the red blood cells with BD FACS lysing solution (BD Biosciences) and samples analyzed by flow cytometry as described below. Cell concentrations were determined by comparing cellular events to bead events.

### Tumor processing

Tissues were processed as previously described^7^. Briefly, following dissection, tumors were weighed and minced into small fragments, then transferred into C tubes from Miltenyi Biotec containing enzyme digest mix with 250U/mL collagenase IV (Worthington Biochemical), 30U/mL DNase I (Millipore-Sigma), 5 mM CaCl2, 5% heat inactivated FBS and HBSS. Tissue was dissociated using a GentleMACS tissue dissociator from Miltenyi Biotech. This was followed by incubation at 37 °C for 30 min with agitation. Enzymatic reactions were quenched using ice cold RPMI containing 10% FBS and 2 mM EDTA. Single cell suspensions were then filtered through 100 µm nylon cell strainers to remove macroscopic debris and analyzed by flow cytometry.

### Flow cytometry

For staining, 2 × 10^6^ cells were stained with Zombie Aqua Viability Dye (BioLegend) in PBS for 10 min on ice, then Fc receptors were blocked with anti-CD16/CD32 antibodies from BD Biosciences (2.4G2) for an additional 10 min. After centrifugation, the supernatant was removed and cell were stained with a surface antibody cocktail containing in FACS buffer (PBS, 2 mM EDTA, 2% FBS) and Brilliant Stain Buffer Plus from BD Biosciences for 20 min on ice. The following antibodies were purchased from BioLegend; F4/80-PerCP/Cy5.5 (BM8), CD11c-PE/Cy7 (N418), CCR7-PE (4B12), CD90.2-A700 (30-H12), CD19-A700 (6D5), MHC-II-BV421 (M5/114.14.2), CD11b-BV605 (M1/70), CD8α-BV650 (53-6.7), Ly-6C-BV711 (HK1.4) and IL-12 PE (C15.6). CD40-FITC (HM40-3), CD103-APC (2E9), CD24-APC e780 (M1/69) and Granzyme B eFluor450 (NGZB) were obtained from Thermo Fisher Scientific. CD80-PE CF594 (16-10A1), CD45-BV786 (30-F11) and Ki-67 FITC (B56) were purchased from BD Biosciences. All samples were resuspended in FACS buffer and acquired on a BD Fortessa flow cytometer. Data were analyzed using FlowJo software from Tree Star, v10.5.

### Nanostring

Snap frozen tumor or lymph nodes were crushed on liquid nitrogen and RNA was extracted using the RNeasy Micro Kit (Qiagen, Germantown, MD) according to the manufacturer’s instructions. cDNA was synthesized using SuperScript VILO (ThermoFisher) according to the manufacturer’s instructions. Samples were hybridized with NanoString (Seattle, WA) pan-cancer immune profiling probes for 16 h and were subsequently loaded into an nCounter SPRINT cartridge. Raw data were normalized utilizing 5–7 housekeeping genes. Fold changes in gene expression and significance were analyzed using Nanostring nCounter software.

### Statistical analysis

Graphical Illustration and data were created and analyzed using Prism from GraphPad Software (v7.0). Individual data sets were compared using Student’s T-test and analysis across multiple groups was performed using ANOVA with individual groups assessed using Tukey’s comparison. Kaplan Meier survival curves were compared using a log-rank test.

## Results

### Analysis of ICOS regulation following radiation therapy

**Figure 1:**
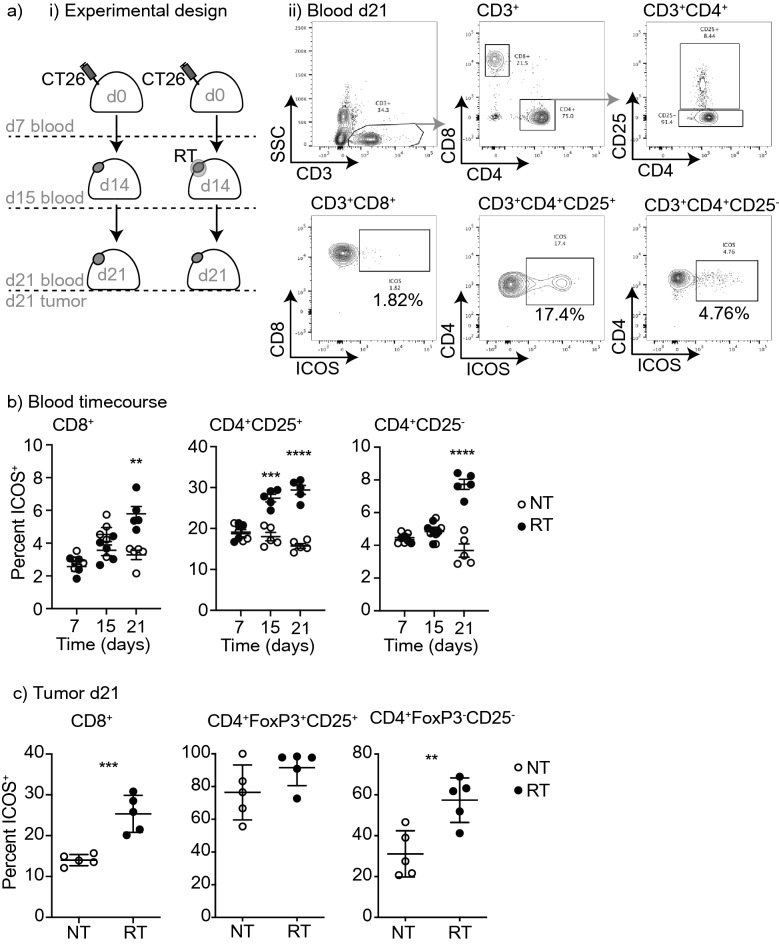
Expression of ICOS on T cell subsets in the blood and tumor following radiation. (**a**) (i) Experimental design. 2 × 10^5^ CT26 cells were implanted subcutaneously into the flanks of Balb/c mice on day 0. Half of the mice received 12 Gy radiation to the tumor using CT guided radiation therapy on day 14. On day 7 (prior to administering radiation), day 15 (24 h post radiation) and day 21 (7 days post radiation) blood was harvested for flow analysis. On day 21 animals were sacrificed and tumors were harvested for flow analysis. (ii) Representative flow plots showing gating strategy for immune cell populations. (**b**) Quantitative dot plot of percent ICOS expression on CD8+, CD4+CD25+(Treg), and CD4+CD25−, in whole blood over time with and without radiation. (**c**) Quantitative dot plots of percent ICOS expression on CD8+, CD4+FoxP3+(Treg), CD4+and in tumors on day 21 with and without radiation. In all graphs, bar represents mean, Error bars SEM, ****p < 0.0001, ***p < 0.001, **p < 0.01, *p < 0.05 as determined by unpaired student *t* test. N = 5 mice per group and experiments were repeated at least 2x.

If radiation therapy can successfully initiate new immune responses to tumor associated antigens, or boost existing T cell responses that can have systemic impact, we hypothesized that this would be reflected in phenotypic changes in circulating T cells in the peripheral blood as a consequence of T cell recirculation^30^. To identify whether ICOS was a potential target on cells activated by radiation therapy, we examined the phenotype of circulating T cells following treatment. CT26 colorectal carcinomas were implanted into immune competent BALB/c mice and allowed to establish for 14 days. Tumors were treated with 12 Gy CT-guided focal radiation to the tumor, avoiding significant dose to draining lymphoid organs. On day 7, 15, and 21 days following tumor implantation (day − 7, + 1, and + 7 relative to radiation) we examined the phenotype of peripheral blood T cell populations using quantitative flow cytometry of whole blood (Fig. 1a). We identified a significant increase in the percent of circulating CD4 T cells that expressed ICOS in the blood 1 and 7 days post radiation therapy. At 1 day following radiation, this increase was predominantly due to increased ICOS expression on the CD4^+^CD25^+^ Treg population (27.42% vs. 18.02%, *p* < 0.0001, n = 5/group), but by day 7 there was increased ICOS expression on non-Treg CD4^+^ T cells in the blood (7.73% vs. 3.68%, *p* < 0.0001, n = 5/group) (Fig. 1b). CD8^+^ T cells similarly demonstrated an increase in ICOS expression in the peripheral blood at day 7 following radiation therapy. The proportion of these cells expressing ICOS was low, suggesting this was not broad, non-specific T cell activation.

To determine whether these changes in ICOS expression in the peripheral blood were also reflected in the tumor, we harvested tumors at day 21 (day 7 following radiation therapy) to evaluate ICOS expression on tumor-infiltrating T cells by flow cytometry. CD4^+^ FoxP3^+^CD25^+^ Tregs were already highly ICOS^+^ at baseline and this proportion did not significantly change following treatment (Fig. 1c). However, there was a significant increase in ICOS expression on non-Treg CD4^+^ T cells in the tumor (62.16% vs. 34.04%, *p* = 0.004, n = 5/group). Similarly, ICOS expression was also increased on CD8^+^ T cells in irradiated tumors (25.34% vs. 14.02%, *p* = 0.007). These data demonstrate that ICOS is upregulated by T cells in the peripheral blood and in the tumor following radiation therapy. While we cannot definitively link these two populations, in patients, ICOS-expressing CD4 non-Treg have been shown to define the proliferating, tumor-specific population of CD4 T cells in the tumor^31^, suggesting radiation therapy increases the presence of antigen-specific cells, or their recognition of antigens. Recent data also demonstrate that ICOS-expressing CD4 are restricted to the tumor stroma, where the majority of MHCII-expressing cells are located^31^, suggesting that this response does not rely on direct antigen presentation by cancer cells, despite the impact of radiation on MHC expression by cancer cells^5^.

**Figure 2:**
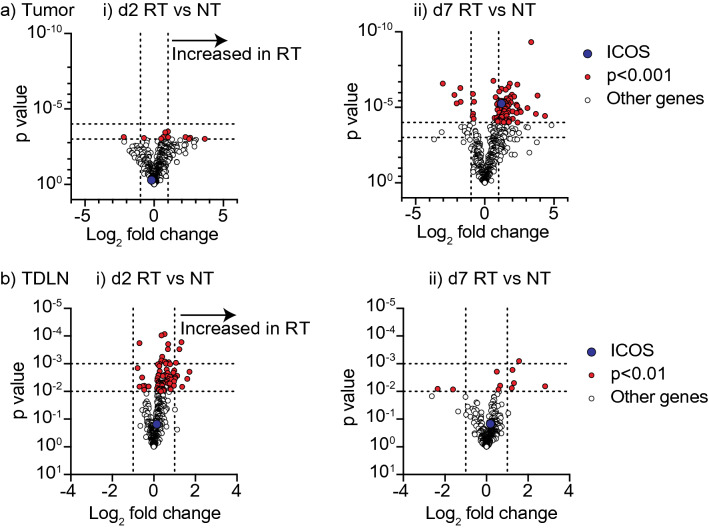
Expression of ICOS in tumor and TDLN following RT. (**a,b**) Panc02-SIY tumors were implanted subcutaneously into flanks of C57BL/6 mice. Tumors were treated with 12 Gy radiation on day 15. Tumors and TDLNs were harvested from untreated (NT) or radiation treated (RT) animals for Nanostring analysis. (**a**) Within the tumor, NT and RT samples were compared to identify differentially expressed genes at (i) day 2 and (ii) day 7 post radiation. Volcano plots were generated using the − log_10_ *p*-value (y-axis) and log_2_ fold change in gene expression (x-axis). Genes were considered significant if the Benjamini–Yekutieli adjusted *p*-value was < 0.1 and the log_2_ fold change was >  ± 1.0. Significant genes, either up or down regulated, are represented as red circles. The ICOS gene is identified as a blue circle and genes that are not significant (not sig) are indicated as white circles. (**b**) The same analysis from (**a**) was used to identify significantly expressed genes between NT and RT samples collected from the TDLN at (i) day 2 and (ii) day 7 post treatment with radiation.

To expand our analysis of ICOS regulation following radiation therapy, we explored alternative datasets. We performed Nanostring analysis of gene expression in Panc02-SIY tumors at day 2 and day 7 following radiation therapy, and similarly analyzed gene expression in their tumor-draining lymph nodes (TDLN) at these time points (Suplementary Tables 1, 2, 3 and 4). Using this dataset, we performed differential gene expression analysis between each of the timepoints for both tumor and TDLN samples and specifically examined ICOS expression. In the tumor, a greater number of genes were significantly altered at day 7 following radiation therapy (370 genes *p* < 0.05) compared to the number that were significantly altered at day 2 (245 genes) (Fig. 2a). Among the genes with increased expression at day 7, ICOS expression is significantly increased at this time point (1.2 log fold increase, *p* < 0.0001) (Fig. 2a), consistent with the flow cytometry. In the TDLN, in contrast to the tumor, we observed that a greater range of genes were regulated at day 2 following radiation therapy (160 genes) than at day 7 following radiation therapy (36 genes), and ICOS was not significantly changed (Fig. 2b). These data may point to a kinetic pattern of T cell response first in the lymphatics and then in the tumor. However, adjusting for multiple comparisons, only the day 7 tumor sample displayed significantly upregulated genes (218 genes *p* < 0.05). To better understand the pattern of response, we identified all T cell-related genes that like ICOS were upregulated at d7 following radiation therapy (Supplementary Fig. 1). These upregulated genes include activation and exhaustion genes such as CD69, Lag3, Tigit, Ctla4, and Pdcd1 (PD1), as well as T cell-related genes that may indicate increased T cell proportions, such as CD8a, CD8b1, and CD3e (Supplementary Fig. 1). Interestingly, none of these were significantly regulated at any other location or timepoint. Some, like CD69 were upregulated at d2 in the tumor, and a number of genes including CD8a and CD8b1 are downregulated in the tumor at d2 (Supplementary Fig. 1); however, these changes were not significant when corrected for multiple comparisons. These data indicate that ICOS expression in the tumor following radiation therapy is co-ordinated with activation and infiltration of T cells in the tumor, and we see no evidence of radiation-mediated alterations in ICOS expression in the lymph node.

### Combination therapy with ICOS antibody and radiation therapy

**Figure 3:**
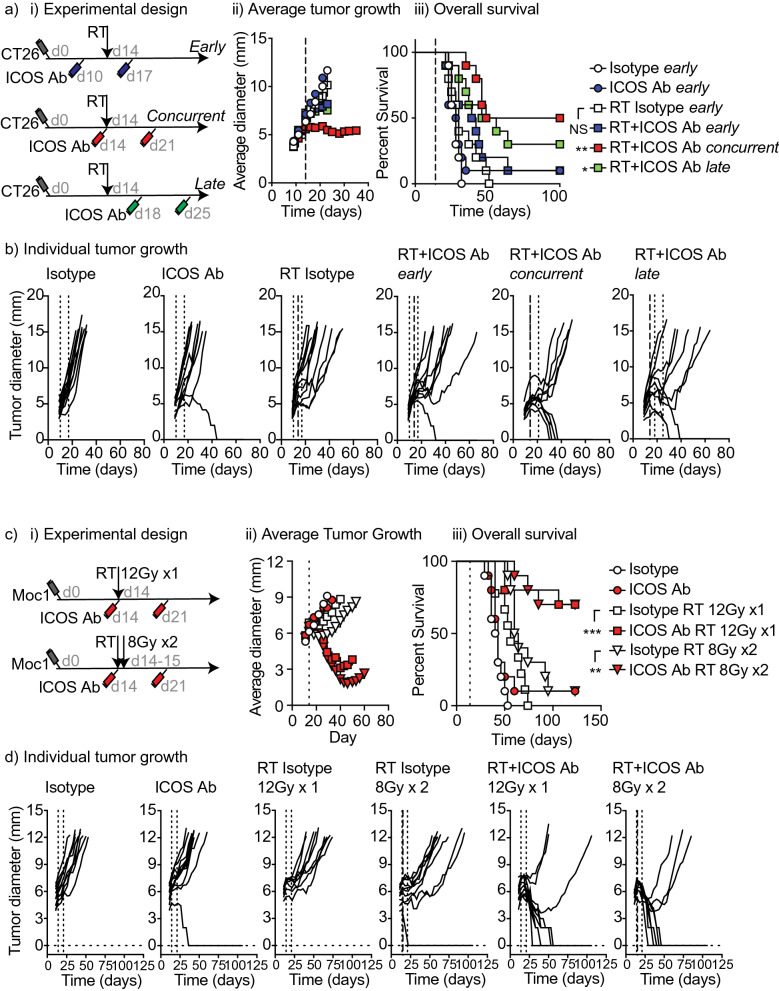
Optimum schedule of administration of ICOS antibody with radiation for overall survival benefit with single fraction and multi-fraction radiation. (**a**) (i) Experimental design. 2 × 10^5^ CT26 cells were implanted subcutaneously in to the flanks of Balb/c mice on day 0. On day 14 all mice in radiation groups were treated with 12 Gy radiation to the tumor using CT guidance. Mice were injected i.p. with 0.25 mg/kg ICOS antibody or in Control groups, mice were treated with equivalent dose of isotype control antibody. For early treatment groups mice were treated with antibody on day 10 and day 17. For concurrent treatment groups mice were treated with antibody on day 14 and day 21. For late treatment groups mice were treated with antibody on day 18 and day 25. (ii) Average tumor growth curves for mice treated as in (i). (iii) Overall survival curves for mice treated as in (i). (**b**) Individual tumor growth curves for mice treated as in (**a**). (**c**) Experimental design. 1 × 10^6^ MOC1 cells were implanted subcutaneously into the flanks of C57Bl/6 mice on day 0. On day 14 all mice in single fraction radiation groups were treated with 12 Gy radiation to the tumor using CT guidance. On days 14 and 15 all mice in dual fraction radiation groups were treated with 8 Gy per fraction radiation to the tumor using CT guidance. Mice were injected i.p. with 0.25 mg/kg ICOS antibody or in Control groups, mice were treated with equivalent dose of isotype control antibody on days 14 and day 21. (ii) Average tumor growth curves for mice treated as in (i). (iii) Overall survival curves for mice treated as in (i). (**d**) Individual tumor growth curves for mice treated as in (**c**). Statistics key for panel (**a**) (iii) and (**c**) (iii): ***p < 0.001, **p < 0.01, *p < 0.05 as determined by Log-rank test. N = 10 mice per group and experiments were repeated at least 2x.

In addition to ICOS, ICOSL is also upregulated in the tumor following radiation therapy (Supplementary Fig. 1). ICOS has been shown to deliver reverse signaling through ICOSL^32^, and delivery of ICOS-Fc has been shown to alter angiogenic regulation via Osteopontin^33^, an alternative partner for ICOSL. Delivery of ICOS-Fc to tumors has been shown to cause altered angiogenesis in the tumor^33,34^. Analysis of angiogenesis-related genes shows evidence of angiogenic remodeling in the tumor environment following radiation therapy (Supplementary Fig. 1). However, given the observation that ICOS is a potential costimulatory molecule that is upregulated on T cells following radiation therapy (Fig. 1), we tested the effect of combining radiation with a novel ICOS agonist antibody^35^. We have previously demonstrated that for optimal tumor control, co-stimulatory antibodies such as OX40 antibody require different timing when compared to blocking antibodies such as anti-CTLA4 relative to the delivery of radiation therapy^36^. For this reason, we tested a range of ICOS antibody treatment timings with the first dose administered before, concurrent with, or following radiation therapy of CT26 tumors (Fig. 3a). We found that concurrent administration was associated with the greatest overall survival (50%) when compared to isotype control (0%), ICOS agonist antibody alone (10%), or radiation plus isotype (0%) (Fig. 3a). However, radiation combined with delivery of the ICOS antibody at any of the treatment times resulted in tumor regression for some proportion of mice. Notably, tumor cures were not related to pre-treatment tumor size, and mice cured of their tumors remained tumor-free long term (Fig. 3b).

To validate these findings in an alternative mouse strain and different tumor model, we treated C57BL/6 mice bearing the Moc1 head and neck carcinoma model^28^ with radiation and concurrent ICOS antibody treatment. As with CT26, the combination of a single 12 Gy radiation dose and ICOS antibody treatment significantly increased overall survival (*p* < 0.0001), with 70% of mice cured of their tumors (Fig. 3c). Alternative hypofractionation regimens have been shown to provide improved responses with anti-CTLA4 therapy^37^, so we evaluated the combination of ICOS antibody with RT delivered as 2 doses of 8 Gy, which is a comparable BED to 1 dose of 12 Gy (28 Gy vs. 26.4 Gy, respectively)^38^. ICOS antibody also increased overall survival to the 8 Gy × 2 alternative fractionation regimen (*p* < 0.005) (Fig. 3c). There was no significant difference in tumor control by either radiation regimen given alone, or either radiation regimen combined with ICOS antibody(Fig. 3d). These data demonstrate that ICOS co-stimulation can improve tumor control in different models in different mouse strains, and with different hypofractionation regimens.

### Mechanism of tumor control

**Figure 4:**
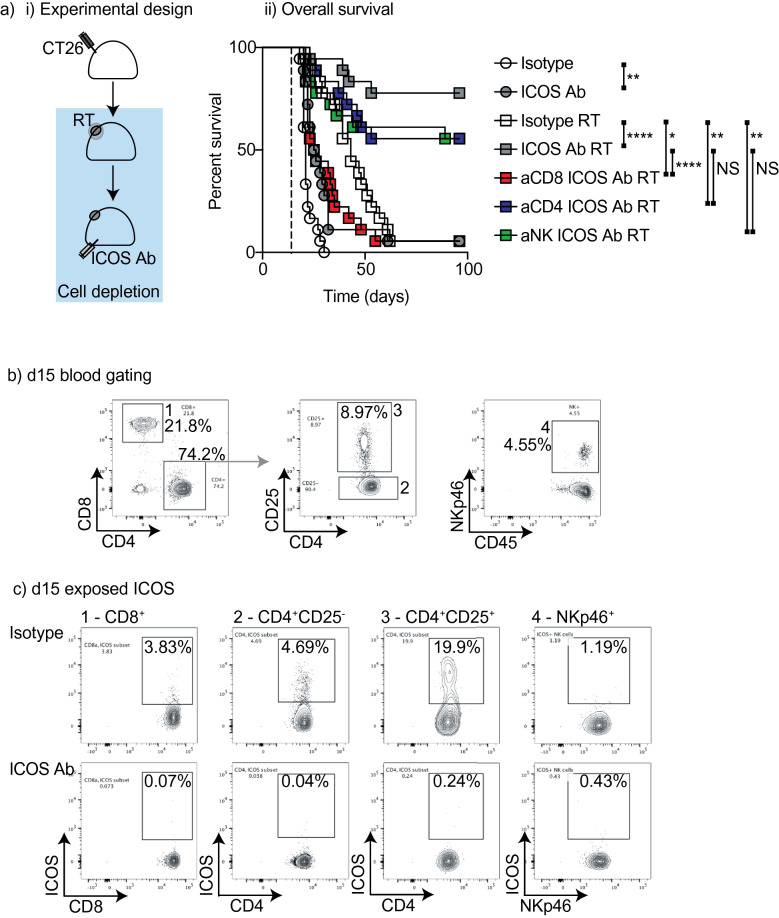
Requirement for ICOS-expressing cell populations to generate survival benefit with RT. (**a**) (i) Experimental design. 2 × 10^5^ CT26 cells were implanted subcutaneously into the flanks of Balb/c mice on day 0. On day 14 all mice in radiation groups were treated with 12 Gy radiation to the tumor using CT guidance. Mice were injected i.p. with 0.25 mg/kg ICOS antibody or in control groups, mice were treated with equivalent dose of isotype control antibody on days 14 and day 21. For depletion groups mice were treated with 250 µg of anti-CD8β, 200 µg anti-CD4(GK1.5), or 100 µl anti-Asialo GM1 (NK depletion) one day prior to radiation and weekly thereafter for duration of animal survival (Blue box indicates duration of depletion). Mice were followed for survival and blood was analyzed weekly by FACS analysis. (ii) Overall survival curves for mice treated as in (i). (**b**) Representative flow plots. (**c**) Flow plots for ICOS expression on relevant cell subsets in ICOS antibody treated or isotype treated groups. ****p < 0.0001, ***p < 0.001, **p < 0.01, *p < 0.05 as determined by Log-rank test. N = 10 mice per group and experiments were repeated at least 2x. For the OS shown in (ii) two experiments were combined for analysis.

Since ICOS is expressed on CD4^+^ T cells, CD8^+^ T cells, and NK cells, we aimed to investigate which cell populations are critical for tumor control by radiation therapy and ICOS antibody. We established CT26 tumors in BALB/c mice and treated them with radiation therapy and concurrent ICOS antibody. Control groups were depleted of CD4^+^, CD8^+^, or NK cells starting 1 day prior to RT. As before, RT plus ICOS antibody resulted in cure in a large proportion of animals (Fig. 4a). Tumor control was not observed in mice depleted of CD8^+^ T cells resulting in significantly reduced overall survival compared to RT plus ICOS antibody (OS RT + ICOS Ab vs. RT + ICOS Ab + aCD8 *p* < 0.0001). In mice depleted of CD4^+^ T cells or NK cells there was a small reduction in the number of mice cured, but not a significant decrease in overall survival (OS RT + ICOS Ab vs. RT + ICOS Ab + aCD4 p = 0.1560; vs. RT + ICOS Ab + aNK *p* = 0.1346). However, in these groups combination treatment was still significantly more effective than RT alone (OS RT + isotype vs. RT + ICOS Ab + aCD4 *p* < 0.01; vs. RT + ICOS Ab + aNK *p* < 0.01). These data suggest that CD4 T cells and NK cells may contribute to anti-tumor response with combination therapy but are not essential for tumor control. By contrast, CD8^+^ T cells are essential for tumor control. Since the density of ICOS expression varies across these cell populations, we examined whether each cell type still retained the agonist antibody bound to their surface. To assess this, we took advantage of the fact that the therapeutic ICOS antibody competitively blocks the binding epitope of the flow cytometry antibody. We discovered a broad loss of detectable ICOS expression at day 15, which is 1 day following the first ICOS antibody dose (Fig. 4b–c). The flow cytometry ICOS antibody was blocked on all of the major cell populations in peripheral blood, including CD8^+^ T cells, Treg, non-Treg CD4^+^ T cells, and NK cells, without detectable loss of these populations. These data suggest that the therapeutic ICOS antibody binds to all of the major lCOS-expressing lymphocyte populations, but only CD8^+^ T cells are essential to cure tumors following radiation therapy and ICOS antibody treatment.

**Figure 5:**
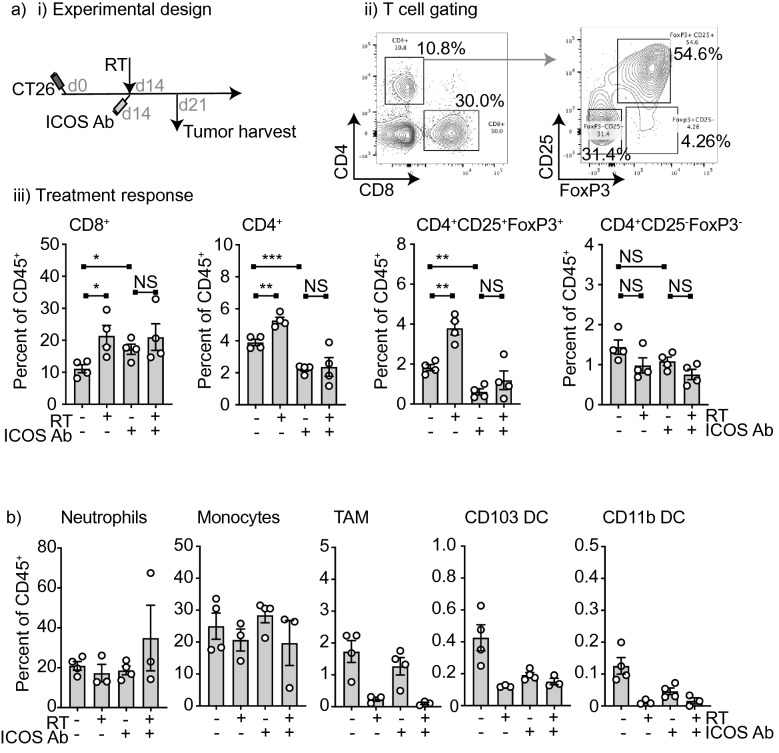
Effect of ICOS antibody on the tumor environment following RT. (i) Experimental design. 2 × 10^5^ CT26 cells were implanted subcutaneously into the flanks of Balb/c mice on day 0. On day 14 all mice in radiation groups were treated with 12 Gy radiation to the tumor using CT guidance. Mice were injected i.p. with 0.25 mg/kg ICOS antibody or in Control groups, mice were treated with equivalent dose of isotype control antibody on day 14 and tumors were harvested on day 21 for FACS analysis. (ii) Representative flow plots. (iii) Quantitative bar graphs of T cell populations of interest in the tumor 7 days following treatment. b. Quantitative bar graphs of myeloid populations of interest in the tumor 7 days following radiation treatment. Error bars SEM, ****p < 0.0001, ***p < 0.001, **p < 0.01, *, *p* < 0.05 as determined by unpaired student *t* test. N = 4 mice per group.

To understand the effect of radiation and ICOS antibody therapy on the tumor immune environment, we harvested tumors 7 days following radiation therapy and analyzed the tumor immune infiltrate by multiparametric flow cytometry(Fig. 5ai,ii). We identified that radiation therapy significantly increased the proportion of CD4^+^FoxP3^+^CD25^+^ T regulatory cells in the tumor following radiation therapy (RT vs. Isotype *p* < 0.01) (Fig. 5aiii) as has previously been described^39,40^. By contrast, ICOS antibody treatment decreased T regulatory cells in the tumor when used as a single agent and also in combination with RT (ICOS Ab vs. Isotype *p* < 0.01; RT + ICOS Ab vs. RT *p* < 0.01), in line with the reported Treg depletion activity of this antibody in preclinical studies^35^. The non-Treg population of CD4^+^ T cells (CD4^+^FoxP3^−^) were not significantly changed in proportion following treatment, and CD8^+^ T cell infiltrates were increased with RT and by ICOS antibody treatment (RT vs. Isotype p < 0.05; ICOS Ab vs. Isotype *p* < 0.05; RT + ICOS Ab vs. RT *p* = 0.0632) (Fig. 5aiii). Next we analyzed the tumors for changes within myeloid populations.The majority of these populations were not significantly altered at 7 days following treatment, though we did notice a decrease in TAM (CD11b^+^CD24^−^Ly6C^-^Ly6G^−^MHCII^+^F4/80^+)^ following radiation therapy, which was independent of ICOS antibody treatment (Fig. 5b).

### Combination of ICOS antibody with radiation therapy and anti-PD1

**Figure 6:**
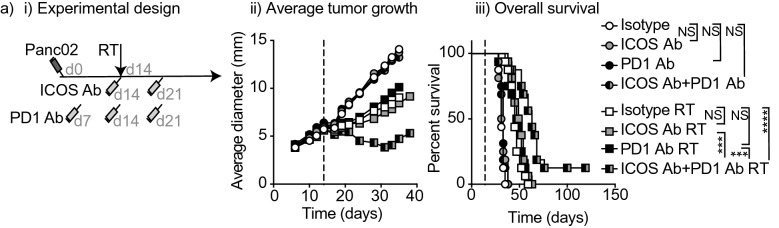
Addition of ICOS antibody to radiation and anti-PD1 on overall survival in the anti-PD1 immunotherapy resistant Panc02 tumor model. (**a**) (i) Experimental design. 2 × 10^5^ Panc02 cells were implanted subcutaneously into the flanks of C57Bl/6 mice on day 0. On day 14 all mice in radiation groups were treated with 12 Gy radiation to the tumor using CT guidance. Mice were injected i.p. with 0.25 mg/kg ICOS antibody or in control groups, mice were treated with equivalent dose of isotype control antibody on day 14 and day 21. For mice in groups receiving PD1 antibody, mice were injected i.p. with 250 µg PD1 antibody on day 7, day 14 and day 21. (ii) Average tumor growth curves for mice treated as in (i). (iii) Overall survival curves for mice treated as in (i). Statistics key for panel (**a**) (iii): ****p < 0.0001, ***p < 0.001, as determined by Log-rank test. N = 10 mice per group and experiments were repeated at least 2x.

Many T cell targeted immunotherapies are more effective when used in combination with other treatments. For example, PD1 blockade has proven to improve most combination immunotherapies preclinically. The CT26 and Moc1 tumor models are known to be relatively responsive to immunotherapy combinations, resulting in high cure rates, and can be effectively cured by radiation plus anti-PD1 without additional combinations^1^. For this reason, we aimed to test combination therapy in a model that is typically poorly responsive to immunotherapy. In our laboratory, the Panc02 pancreatic adenocarcinoma model has proven to be unresponsive to T cell targeted immunotherapy in combination with radiation therapy (data not shown). For this reason, Panc02 is a useful model to evaluate the combination of different immunotherapies with radiation since they are not effective individually. Supporting this rationale, ICOS antibody, anti-PD1, and the combination of the two did not affect tumor growth or overall survival (Fig. 6ai,ii,iii). In addition, neither ICOS antibody nor anti-PD1 combined with radiation increased overall survival compared to radiation combined with the isotype control. However, the full combination of radiation, ICOS antibody, and anti-PD1 significantly increased overall survival compared to any other radiation treatment (RT + ICOS Ab + aPD1 vs. RT + ICOS Ab *p* < 0.001; vs. RT + aPD1 *p* < 0.001; vs. RT + isotype *p* < 0.0001) and compared to all antibody treatments in the absence of radiation therapy (all *p* < 0.001) (Fig. 6ai,ii,iii). These data demonstrate that anti-ICOS can be layered with anti-PD1 to improve outcome even in recalcitrant tumors.

## Discussion

These studies demonstrate that, in mouse models, ICOS is upregulated on T cells in the peripheral blood and tumor following radiation therapy, and that when given at the optimal timing, the combination of ICOS agonism and radiation can result in cures in tumor bearing mice. The mechanism of tumor eradication is dependent on CD8^+^ T cells, and is effective in a range of preclinical tumor models and across different radiation fractionation schemes. Importantly, our data indicate that, at least in preclinical models, ICOS antibody can be combined with clinically effective therapies such as anti-PD1 to improve tumor control where they are otherwise ineffective.

We compared a range of timing for the combination of ICOS antibody and radiation therapy and identified that while improvement was seen with the combination treatment across all the different timing parameters tested, concurrent administration resulted in the highest number of cured animals. The ICOS antibody used in this study has agonistic activity resulting in T cell activation and can also mediate Treg reduction in the tumor microenvironment in preclinical models, likely due to antibody-dependent cell-mediated cytotoxicity^35^. Notably, the timing of response matched our experience with OX40 antibody which is optimally delivered immediately following radiation therapy, rather than anti-CTLA4, which is optimally delivered prior to radiation^36^. These data are more consistent with the ICOS antibody having a costimulatory mechanism of action on cognate antigen-stimulated T cells, as observed with OX40 antibody^36^. Such a mechanism would demand that the agonist antibody is available during this induction of ICOS to optimally stimulate the tumor-reactive cells that we observed following radiation. The initial increase in ICOS^+^ cells was first identified in T regulatory cells in the peripheral blood 24 h after radiation. Other T cells exhibited increased ICOS expression at day 7 following radiation. This data may be important since 24 h following radiation therapy is likely too early for antigen-driven expansion in the draining lymph node mediated by cancer cell death in the treatment field to be reflected in the peripheral blood. According to dogma, such an event would require cancer cell death in the tumor environment, dendritic cell mediated trafficking of antigen and cross-presentation in the draining lymph node, and cognate T cell proliferation before these cells are released from the lymph node^30^. The combination of these events may require days before newly activated cells reach systemic circulation^30^. This delayed timeline more closely matches the response of CD8^+^ T cells and non-Treg CD4^+^ T cells, so for this reason we believe that the mechanisms by which Treg versus non-Treg CD4^+^ T cells and CD8^+^ T cells increase their ICOS expression are different. While we did not observe upregulation of ICOS in the TDLN or tumor at early time points following treatment with radiation therapy, ICOS is expressed at baseline in both the untreated tumor and in T cells in the lymph node^31,41^. Therefore the ICOS antibody may be functioning in either site independent of radiation-mediated regulation. The identity of the ICOS-expressing cells in the tumor remains to be determined. ICOS-expressing CD4 T cells in patient tumors have been shown to display the transcription factor markers of Th1, Th2, Th17, and Tfh cells^31^. However, Tfh are generally associated with ectopic lymphoid follicles^42,43^, and while we have observed these in patients^44^, our preclinical models do not develop lymphoid follicles over the time-course of these experiments. Th2 and Th17 CD4 T cells are not always considered a positive feature of tumor environments^45,46^; however, patients with tumors expressing increased proportions of ICOS-expressing CD4 T cells have an improved prognosis^31^. Thus, our data are consistent with ICOS being an important target to identify both CD8 and non-Treg CD4 T cells with the potential to participate in tumor control.

Not surprisingly, CD8^+^ T cells were found to be absolutely necessary for control of residual tumors. While depletion of other populations including CD4 and NK cells resulted in partial abrogation of tumor control, the depletion of either of these populations alone was not sufficient to eliminate the response of combined therapy. While we did not test the effect of CD8, CD4, or NK depletion on untreated tumors or radiation alone, our prior work has demonstrated that untreated or irradiated CT26 tumors are not impacted by CD4 depletion^36^. While ICOS antibody is dependent on the Fc region^35^, like anti-CTLA4 or anti-CD40 antibodies^47–49^, myeloid expression of FcR is more critical for the function of these antibodies in preclinical models than the presence of NK cells^48^. Our cell depletion data may imply that while CD4^+^ T cells, NK cell, and CD8^+^ T cells each enhance the anti-tumor response, there may be some overlapping redundancy for the CD4^+^ T cells and NK cells, and that the presence of one of these cell populations may partially make up for the absence of one of the other populations. However, CD8^+^ T cells serve as a common, essential pathway for eradication of residual tumor in these studies.

When analysing the tumor environment following radiation alone we observed an increase in Tregs in the tumor environment. This is consistent with previously published results that showed increases in both the number and function of regulatory T cells following radiation^39,40^. Treatment with ICOS antibody alone has previously been shown to result in regulatory T cell depletion in the tumor environment in preclinical studies^35^, and consistent with those findings, we show significantly fewer regulatory T cells in the tumor environment with ICOS antibody alone. Interestingly ICOS antibody was able to abrogate the significant increase in regulatory T cells associated with tumor radiation and thus negates one of the major negative regulatory mechanisms that occurs following radiation^39,40,50^. It should be noted, however, that the ability of antibodies to efficiently deplete target cells relies on many factors, which often does not translate from mouse to human, as reported in the case of Treg depletion by anti-CTLA4 antibodies^51,52^.

These studies demonstrate that ICOS is induced on both CD4 and CD8 T cells in the peripheral blood and the tumor following tumor radiation and demonstrated that targeting this pathway with a novel ICOS agonist antibody can enhance immune mediated local control following radiation. Timing studies demonstrating that concurrent radiation and ICOS agonist antibody results in the highest rate of tumor control provide a starting point for clinical trial design^53^. These data provide a promising rationale for translational studies in patients looking at the combination of radiation and ICOS agonism in enhancing tumor control and increasing organ preservation in clinical scenarios where radiation is used for tumor control and cure. Moreover, tumors that were unresponsive to radiation therapy and anti-PD1 could be cured by the addition of ICOS antibodies as a triple therapy. This has significant implications to improve the disappointing response rates to the combination of radiation therapy and PD1 blockade in patients.

## Supplementary Information

Supplementary Information 1.

Supplementary Information 2.

Supplementary Information 3.

Supplementary Information 4.

Supplementary Information 5.

## Data Availability

All data generated or analysed during this study are included in this published article [and its supplementary information files] or available from the corresponding author on reasonable request.
