# Supplementary material for: ICOS is upregulated on T cells following radiation and agonism combined with radiation results in enhanced tumor control
Source: Sci Rep. 2022 Sep 2;12:14954. doi: 10.1038/s41598-022-19256-8 (PMC9440216; doi:10.1038/s41598-022-19256-8)
Supplement: Supplementary file 5 — Supplementary Information 5. [file 41598_2022_19256_MOESM5_ESM.pdf]

Supplementary Figure 1

i) ICOS pattern gene expression

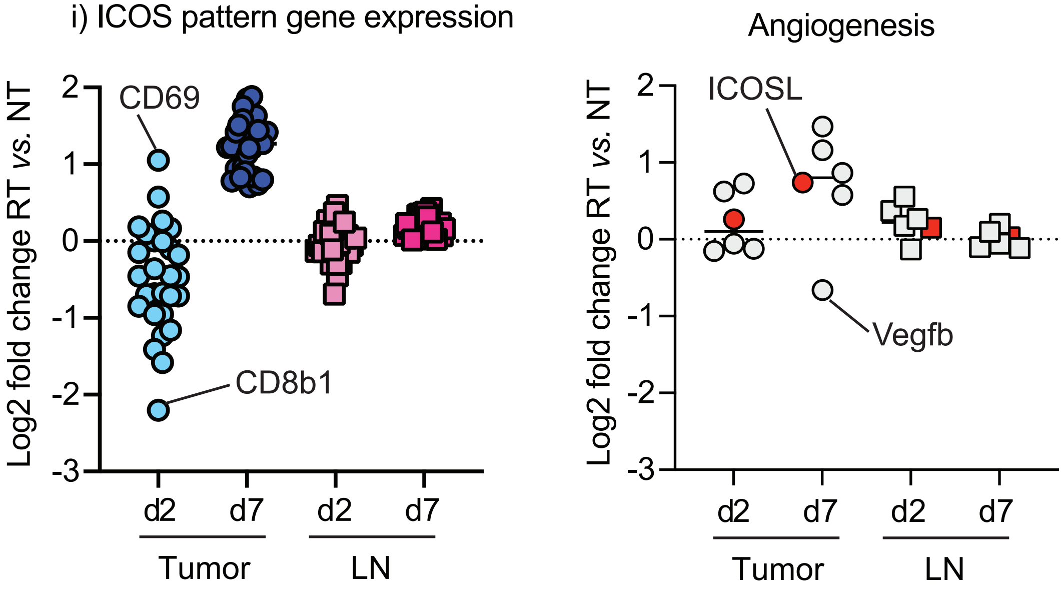

ii) Represented gene list

Gene	Tu d2 Log2 fc	BY.p.value	Tu d7 Log2 fc	BY.p.value	LN d2 Log2 fc	BY.p.value	LN d7 Log2 fc	BY.p.value
Cd28-mRNA	-0.458	1	1.88	0.000641	-0.0253	1	0.341	1
Il7r-mRNA	-0.107	1	1.85	0.000581	0.00338	1	0.422	1
Eomes-mRNA			1.75	0.00124	-0.0416	1	0.0484	1
Cxcr6-mRNA	-1.58	0.216	1.63	0.000741	-0.13	1	0.075	1
Fasl-mRNA	-0.676	0.95	1.6	0.000697	-0.0546	1	0.353	1
Tigit-mRNA	-0.715	0.6	1.58	0.00743	-0.69	0.149	0.343	1
Cd8b1-mRNA	-2.2	0.216	1.57	0.000581	-0.294	1	0.207	1
Pdcd1-mRNA	-0.952	0.74	1.51	0.0141	-0.304	1	0.0546	1
Lag3-mRNA	-0.147	1	1.44	0.00625	-0.454	0.374	0.339	1
Il2rb-mRNA	-0.454	1	1.42	0.0102	-0.0324	1	0.377	1
Cd8a-mRNA	-1.23	0.263	1.42	0.000641	-0.254	1	0.0771	1
Thy1-mRNA	-0.466	0.27	1.42	0.000641	-0.0607	1	0.0998	1
Cd3e-mRNA	-1.41	0.23	1.42	0.000641	-0.298	1	0.185	1
Cd2-mRNA	-0.685	0.488	1.34	0.000641	0.264	0.685	0.108	1
Cd247-mRNA	-0.713	0.769	1.27	0.000825	-0.145	1	0.236	1
Cxcr4-mRNA	0.00374	1	1.23	0.000674	0.461	0.432	0.134	1
Ly9-mRNA	-0.7	0.246	1.22	0.000641	0.071	1	0.0684	1
Cd69-mRNA	1.05	0.459	1.21	0.0438	0.249	1	0.0153	1
**Icos-mRNA**	**-0.181**	**1**	**1.2**	**0.000641**	**0.146**	**1**	**0.192**	**1**
Zap70-mRNA	-0.848	0.365	1.13	0.00358	-0.14	1	0.111	1
Il2rg-mRNA	0.0989	1	0.968	0.00356	0.18	0.336	0.163	1
Ccr5-mRNA	0.572	0.298	0.942	0.00346	-0.0554	1	0.365	1
Lck-mRNA	-0.955	0.246	0.903	0.0228	0.0377	1	0.0362	1
Cd3d-mRNA	-1.16	0.23	0.835	0.0213	0.0878	1	0.0231	1
Entpd1-mRNA	0.187	1	0.829	0.0389	0.368	0.149	0.229	1
Gata3-mRNA	-0.361	1	0.797	0.0329	-0.115	1	0.117	1
Ctla4-mRNA	-0.00529	1	0.783	0.0136	0.124	1	0.313	1
Icosl-mRNA	0.26	1	0.739	0.0111	0.151	1	0.0284	1
Cxcl16-mRNA	0.163	1	0.706	0.00269	0.0408	1	0.16	1
Vegfb-mRNA	-0.122	1	-0.662	0.0126	-0.132	1	-0.103	1
Vegfc-mRNA	-0.0566	1	1.16	0.233	0.367	1	0.201	1
Vegfa-mRNA	0.621	0.442	0.864	0.0719	0.175	1	-0.0582	1
Pecam1-mRNA	-0.153	1	0.577	0.0286	0.265	0.985	-0.113	1
Angpt2-mRNA	0.727	0.731	1.47	0.123	0.555	1	0.0982	1
